# Adrenal Hormones in Common Bottlenose Dolphins (*Tursiops truncatus*): Influential Factors and Reference Intervals

**DOI:** 10.1371/journal.pone.0127432

**Published:** 2015-05-18

**Authors:** Leslie B. Hart, Randall S. Wells, Nick Kellar, Brian C. Balmer, Aleta A. Hohn, Stephen V. Lamb, Teri Rowles, Eric S. Zolman, Lori H. Schwacke

**Affiliations:** 1 National Oceanic and Atmospheric Administration, National Ocean Service, National Centers for Coastal Ocean Science, Hollings Marine Laboratory, Charleston, South Carolina, United States of America; 2 Chicago Zoological Society, c/o: Mote Marine Laboratory, Sarasota, Florida, United States of America; 3 National Oceanic and Atmospheric Administration, National Marine Fisheries Service, Southwest Fisheries Science Center, La Jolla, California, United States of America; 4 National Oceanic and Atmospheric Administration, National Marine Fisheries Service, Southeast Fisheries Science Center, Beaufort, North Carolina, United States of America; 5 Animal Health Diagnostic Center, Cornell University College of Veterinary Medicine, Ithaca, New York, United States of America; 6 National Oceanic and Atmospheric Administration, National Marine Fisheries Service, Office of Protected Resources, Silver Spring, Maryland, United States of America; Sonoma State University, UNITED STATES

## Abstract

Inshore common bottlenose dolphins (*Tursiops truncatus*) are exposed to a broad spectrum of natural and anthropogenic stressors. In response to these stressors, the mammalian adrenal gland releases hormones such as cortisol and aldosterone to maintain physiological and biochemical homeostasis. Consequently, adrenal gland dysfunction results in disruption of hormone secretion and an inappropriate stress response. Our objective herein was to develop diagnostic reference intervals (RIs) for adrenal hormones commonly associated with the stress response (i.e., cortisol, aldosterone) that account for the influence of intrinsic (e.g., age, sex) and extrinsic (e.g., time) factors. Ultimately, these reference intervals will be used to gauge an individual’s response to chase-capture stress and could indicate adrenal abnormalities. Linear mixed models (LMMs) were used to evaluate demographic and sampling factors contributing to differences in serum cortisol and aldosterone concentrations among bottlenose dolphins sampled in Sarasota Bay, Florida, USA (2000–2012). Serum cortisol concentrations were significantly associated with elapsed time from initial stimulation to sample collection (p<0.05), and RIs were constructed using nonparametric methods based on elapsed sampling time for dolphins sampled in less than 30 minutes following net deployment (95% RI: 0.91–4.21 µg/dL) and following biological sampling aboard a research vessel (95% RI: 2.32–6.68 µg/dL). To examine the applicability of the pre-sampling cortisol RI across multiple estuarine stocks, data from three additional southeast U.S. sites were compared, revealing that all of the dolphins sampled from the other sites (N = 34) had cortisol concentrations within the 95th percentile RI. Significant associations between serum concentrations of aldosterone and variables reported in previous studies (i.e., age, elapsed sampling time) were not observed in the current project (p<0.05). Also, approximately 16% of Sarasota Bay bottlenose dolphin aldosterone concentrations were below the assay’s detection limit (11 pg/mL), thus hindering the ability to derive 95th percentile RIs. Serum aldosterone concentrations from animals sampled at the three additional sites were compared to the detection limit, and the proportion of animals with low aldosterone concentrations was not significantly different than an expected prevalence of 16%. Although this study relied upon long-term, free-ranging bottlenose dolphin health data from a single site, the objective RIs can be used for future evaluation of adrenal function among individuals sampled during capture-release health assessments.

## Introduction

As inhabitants of a dynamic environment, bottlenose dolphins (*Tursiops truncatus*) are exposed to a variety of stressors. These include essential biological demands that accompany processes such as growth, metabolism, reproduction, predator avoidance, and social interaction, as well as degradation of habitat from increasing industrialization and coastal development. Mammalian endocrine systems have adapted to accommodate the intrinsic stressors of growth and reproduction, while cetaceans have developed further biological modifications to cope with different metabolic and physiological requirements that accompany life in a marine environment [1, 2]. However, anthropogenic sources of stress (e.g. boat traffic, entanglement, harassment, chemical contamination, noise) present entirely new challenges to which wild dolphin populations may not be able to acclimate.

The mammalian endocrine gland primarily responsible for mediating the effects of chronic and acute stressors is the adrenal gland. Mammalian adrenal glands are responsible for the secretion and release of mineralocorticoids (e.g. aldosterone) and glucocorticoids (e.g. cortisol) to regulate and maintain electrolyte homeostasis and cope with the physiological response to external and internal stressors. Aldosterone helps to control sodium balance, which is especially critical for marine mammals. Cortisol is the primary glucocorticoid secreted by the adrenal gland, under the mediation of adrenocorticotropic hormone (ACTH) from the pituitary. When exposed to a particular stressor, the endocrine and other organ systems undergo a cascade of events that involves the release of ACTH by the pituitary and renin by the kidney, prompting an increased secretion of cortisol and aldosterone, respectively, by the adrenal gland [2].

Previous studies have examined cetacean adrenal response to experimental stressors such as capture and restraint, captivity, and cold temperatures [3–6], as well as hormonal variation due to season, sex, age, elapsed sampling time, and time of day [6–8]. Results from these studies have revealed that adrenal hormones are influenced to some extent by season, time of day, and sampling duration [6–9]. In most of these studies, differences in mean concentrations relative to these factors were examined, but in order to extrapolate their findings, the distribution of hormones among other populations would need to be the same. As Schwacke et al. (2009) point out, mean values can be similar between populations with different underlying distributions (or vice versa), but the detection of clinically relevant abnormalities often relies on the ability to determine differences in the tails of a distribution [10, 11]. The methods described herein address these differences in the distribution tails. Reference intervals (RIs) are commonly used by veterinarians and physicians to clinically evaluate the health of individuals. Objectively evaluated and constructed from large datasets of randomly sampled individuals, RIs are considered to be representative of a normal population. Therefore, analyte values above or below the threshold of a RI are considered to be unusual and often coincide with a health condition or disease state (e.g. low hemoglobin and anemia [12]).

The overall objective of this study was to establish bottlenose dolphin adrenal hormone RIs that can be used to assist in evaluating an individual’s endocrine health during capture-release health assessment projects. Because the endocrine response can be influenced by extrinsic (e.g. sampling duration, time of day) and intrinsic (e.g. age, sex) factors, relationships between these variables and hormone concentrations were evaluated to refine the precision of the RIs. Finally, since the RIs relied on data acquired from a single sampling site, bottlenose dolphin adrenal hormone concentrations from three other southeastern U.S. sites were compared to the RIs to evaluate their applicability to other estuarine stocks. Our analyses contribute to a broader understanding of the normal variation for health parameters in wild dolphins that is essential to compare and evaluate individual and population health [11, 13–16].

## Materials and Methods

### Ethics Statement

All sampling was completed under research permits issued by the National Marine Fisheries Service (Sarasota: 522–1569, 522–1785, 15543; Georgia: 932-1905/MA-009526; St. Joseph Bay, FL: 932-1489-05; Beaufort, NC: 779-1681-00). Research was approved by the Mote Marine Laboratory and the National Oceanic and Atmospheric Administration’s National Marine Fisheries Service Institutional Animal Care and Use Committees, and was performed in accordance with ASM guidelines for research on live mammals [17].

### Bottlenose Dolphin Capture-Release Health Assessments

Bottlenose dolphins inhabiting Sarasota Bay, FL, USA, have been the subject of population and health studies since the 1970s [18]. Health assessments involving temporary capture have been conducted since 1988 and have been previously described [16, 18]. Dolphins were encircled in shallow water with a 500m x 4m seine net [19], restrained by trained personnel, briefly examined by experienced veterinarians, and a blood sample was collected prior to lifting the dolphin onto a padded and shaded sampling platform for the collection of morphometrics and biological samples including urine, feces, gastric fluid, milk, microbiological swabs, skin, and/or blubber [16]. Sampled animals’ ages were determined primarily through long-term field observations or, in a few cases, from sectioning of a tooth [20], which was obtained under local anesthesia during onboard sampling. Following sampling, dolphins were moved back into the water and released. In some cases, depending upon specific research objectives, a second blood sample was collected immediately prior to release. The blood sample collected before and following onboard sampling will be hereafter referred to as “pre-sampling” and “post-sampling”, respectively.

### Bottlenose Dolphin Blood Collection and Processing

Methods for bottlenose dolphin blood collection and processing have been described previously [11, 21]. Briefly, blood was collected via catheterization of the ventral vasculature of the dolphin fluke. Blood samples for hormone analyses were collected in serum separator tubes and centrifuged to separate serum from red blood cells. Aliquots of the sera were frozen and shipped overnight to Cornell University’s Animal Health Diagnostic Center’s Endocrinology Laboratory (Ithaca, NY, USA) for analysis.

Cortisol was measured using a competitive chemiluminescent immunoassay which uses reagents specifically designed for the automated IMMULITE system (Siemens Healthcare Diagnostics, Los Angeles, CA). The calibration range is 1 to 50 ug/dL and analytical sensitivity is 0.2 ug/dL. Aldosterone was measured using a solid-phase radioimmunoassay Coat-A-Count reagents (Siemens Healthcare Diagnostics, Los Angeles, CA) designed for quantification of aldosterone in unextracted serum or heparin samples. The assay calibration range is 25 to 1,200 pg/mL and the analytical sensitivity is 11 pg/mL. The process for assay validation and intra-assay variability for both cortisol and aldosterone has been previously reported [22].

### Experimental Design and Analysis

The Sarasota Bay bottlenose dolphin capture-release dataset represents over three decades of sampling with repeated measures of many individuals. The 2000–2012 sub-dataset used for cortisol analyses included 189 pre-sampling observations, representing 118 individuals (60 males, 58 females; Table 1; S1 Dataset). Because of differences in funding and research priorities between health assessment projects, post-sampling blood was only collected between 2000 and 2005; therefore, a smaller dataset was used for post-sampling cortisol analyses (Table 1; S1 Dataset). Similarly, aldosterone was not measured for each individual in the pre-sampling dataset, so pre-sampling aldosterone analyses included 162 observations from 103 individuals (Table 1) and post-sampling analyses were not conducted due to a limited sample size (N = 31). Data were restricted to May-July, when most of the sampling occurred, to limit potential variation due to season [8, 9], as well as the years 2000–2012 to maintain laboratory consistency since different diagnostic laboratories were used for analyses prior to 2000 [21]. No samples were collected in 2007 as health assessments were not conducted that year. Inter-annual variation in cortisol and aldosterone was evaluated using a Kruskal-Wallis test with post-hoc pairwise comparisons, which determined sporadic differences between years not attributable to any known sampling or environmental biases (R. Wells, pers. comm.). Therefore, data from all years were included for analyses. Pregnant animals (N = 11), diagnosed by ultrasonography, were excluded from analyses due to hormonal fluctuations coincident with pregnancy [8]. The ages of dolphins sampled ranged from 2–50 years (males: 2–43; females: 2–50), providing an opportunity to examine hormone concentrations across a broad age range. Animals of unknown age (N = 3) were excluded from analyses. Dolphins with small calves (estimated to be <2 yrs age) were avoided for capture; therefore, analyses did not include very young animals. All blood samples were collected between the hours of 8:00AM and 4:19PM, U.S. Eastern Standard Time. An elapsed sampling time covariate was calculated as the difference between the time of net deployment and venipuncture for pre- or post-sampling blood collection [11], and varied among dolphins. Elapsed times for pre-sampling blood draws ranged from 8 to 73 min, with a median of 22 minutes, and the post-sampling elapsed time range was 90 to 273 min, with a median of 130 minutes. Pre-sampling observations without information on sampling time (N = 1) or with an extreme outlier for elapsed time (N = 1) were excluded from analyses.

**Table 1:** Descriptive Statistics.

Variable	Class	N_i_	Mean	SD	Median	Range	% BDL* ^+^
**Pre-Sampling**							
**Cortisol**	All	118	2.53	1.08	2.48	0.10–6.50	0.85
	Females	58	2.61	1.03	2.72	0.10–4.42	1.72
	Males	60	2.46	1.14	2.34	0.75–6.50	0.00
	Adult	56	2.82	0.97	2.88	1.10–6.50	0.00
	Subadult	62	2.27	1.12	2.19	0.10–4.69	1.61
**Post-Sampling**							
**Cortisol**	All	52	4.27	1.17	4.08	1.84–7.30	0.00
	Females	28	4.12	1.10	4.02	2.22–6.84	0.00
	Males	24	4.44	1.24	4.49	1.84–7.30	0.00
	Adult	30	4.46	0.98	4.36	2.81–7.30	0.00
	Subadult	22	4.01	1.37	3.84	1.84–6.84	0.00
**Aldosterone**							
	All	103	83.57	92.56	51.62	5.50–492.00	15.53
	Females	50	91.03	103.13	49.90	5.50–492.00	16.00
	Males	53	76.52	81.72	56.14	5.50–467.00	15.09
	Adults	46	57.97	57.18	42.13	5.50–230.00	21.74
	Subadult	57	104.22	109.56	76.14	5.50–492.00	10.53

Descriptive statistics for hormone data for bottlenose dolphins sampled in Sarasota Bay, FL (2000–2012). Abbreviations: N_i_ = number of individuals; “% BDL” indicates the percentage of observations with hormone concentrations below respective detection limits.

*Values below detection limit calculated as ½ of the detection limit for the assay (Schwacke et al. 2009)

^+^Detection Limits: cortisol (0.2 μg/dL); aldosterone (11 pg/mL)

R 2.15.3 (R Foundation for Statistical Computing, 2010) and SAS 9.3 (SAS Institute, 2003) were used for statistical analyses. Descriptive statistics and tests for normality (i.e. Shapiro—Wilks) were conducted for cortisol and aldosterone, as well as all potential covariates. Also, the percentage of observations with hormone concentrations below respective assay detection limits (BDL; cortisol: 0.20 μg/dL; aldosterone: 11pg/mL) was calculated. For BDL measurements, hormone concentrations were computed as half of the detection limit (i.e. cortisol: 0.10 μg/dL; aldosterone: 5.50 pg/mL) [11]. Finally, based on findings from previous studies [4, 6, 23], a Spearman’s correlation test was used to examine the association between cortisol and aldosterone using the earliest observation for dolphins sampled in multiple years between 2000 and 2012.

### Assessment of Influential Factors

Health parameter measurements may be influenced by sampling factors related to an individual (e.g. age, sex, time), thereby necessitating RIs that are partitioned by these variables. Using the nlme package in R and restricted maximum likelihood estimation methods [24], two linear mixed models (LMM) were used to explore influential demographic or sampling factors for pre-sampling cortisol concentrations while accounting for repeated measurements of some dolphins across years: 1) one for all sampled dolphins which evaluated associations with all covariates; 2) one for adult females (i.e. ≥10 years of age; [25]) that included lactation status in addition to the other factors. Similarly, LMMs were used to explore variation in post-sampling cortisol and pre-sampling aldosterone for all dolphins that were sampled for these analyses 2000–2012. Covariates for the LMMs were based on previously reported correlates and known biological functions and included elapsed sampling time, lactation status, sex, age, and sampling time of day as fixed effects [6–8, 23], while individual ID was a random effect. Significant associations were identified using α = 0.05. Because of the high percentage of BDL aldosterone (approx. 16%, Table 1), aldosterone concentrations were log-transformed for LMM development.

### Cortisol Reference Intervals

A nonparametric bootstrap method was used to estimate 95^th^ percentile RIs and corresponding 90% confidence intervals for pre and post-sampling cortisol [11, 26]. Significant factors identified by the cortisol LMM were considered as potential variables for partitioning the RI. The need to partition [26] was evaluated by statistically comparing differences between strata using bootstrap methods and confidence interval calculations according to procedures described by Schwacke et al. (2009). More specifically, the 2.5^th^ and 97.5^th^ percentile of values from each stratum (i.e. pre-sampling <30 minutes elapsed time and post-sampling) were sampled with replacement for a total of 1000 iterations. The difference in values between the strata for each percentile was calculated, and the non-parametric 95^th^ percentile range for the 1000 differences was computed. If the 95^th^ percentile range for the difference did not contain 0, then the mean difference between strata was considered statistically significant and supported data partitioning [11]. RI calculations only included the first observation for dolphins that were repeatedly sampled between 2000 and 2012 because presumably dolphins would be less sensitized to capture. Extreme outliers were evaluated using Grubbs’ test and omitted for RI estimation as appropriate [26]**.** The fit of these RIs was evaluated by examining plots of the original hormone concentrations and reference thresholds.

### Application

The cortisol RIs were based on data from a single sampling site. To examine the applicability of the pre-sampling cortisol RIs across a wide geographic range and among putatively different inshore stocks, pre-sampling cortisol data from previous capture-release health assessments (Beaufort, NC [11]; Sapelo/Brunswick, GA, [15]; and St. Joseph Bay, FL, [13]) were compared to the RIs. The prevalence of out-of-range observations was calculated for each site and compared to an expected prevalence (i.e. 2.5%), given the number of dolphins sampled. Similarly, assuming an expected prevalence of 16% for BDL aldosterone concentrations, the proportion of dolphins with BDL aldosterone was calculated for each previous assessment to evaluate the detection limit as a reasonable threshold for identifying animals with low aldosterone.

## Results and Discussion

### Cortisol

**Fig 1:**
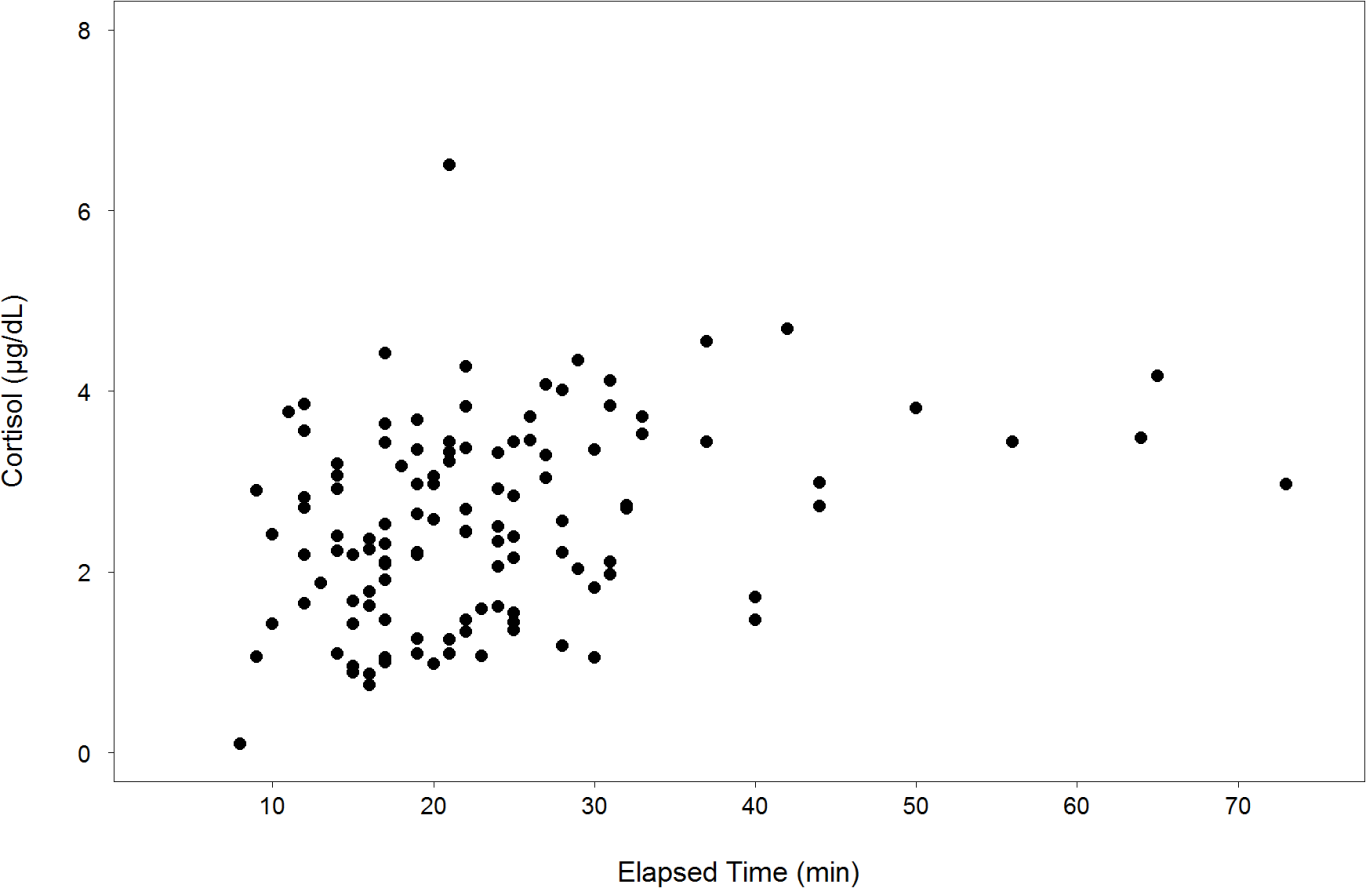
Pre-Sampling Cortisol versus Elapsed Sampling Time. Pre-sampling cortisol concentration (μg/dL) vs. elapsed sampling time (min) for bottlenose dolphins sampled in Sarasota Bay, FL 2000–2012 *(N = 118)*.

Plots of our pre-sampling data revealed significant variation up to approximately 30 minutes elapsed, after which levels were generally higher, although the number of pre-samples collected after 30 minutes elapsed was limited (Fig 1). These results are consistent with findings from Orlov et al. (1988) where peak cortisol concentrations were achieved in a Black Sea bottlenose dolphin (*T*. *truncatus ponticus*) within 30–45 minutes following a stressful event. Furthermore, the mean cortisol concentration for post-samples was significantly higher than pre-samples (Paired t-test, t = 13.32, df = 51, p<0.0001), which is consistent with findings from Fair et al. (2014) where significant elevations in cortisol concentrations were observed between pre-sampling (mean elapsed time = 22 minutes) and post-sampling (mean elapsed time = 97 minutes) serum samples.

Elapsed sampling time was the only variable with significant influence on pre-sampling serum cortisol concentrations (LMM, p<0.05; Table 2). For post-samples, elapsed time was not significantly associated with cortisol concentrations (LMM, p = 0.43; Table 2). Age, sex, lactation status, and time of day were not significantly associated with pre or post-sampling cortisol (p≥0.05; Table 2). These results are in concurrence with previous studies that did not find differences in serum cortisol concentrations between sexes or age classes [7, 22]. However, as opposed to other studies that have observed small but statistically significant changes in circulating cortisol throughout the day [8, 23, 27], our study did not find an association with time of day for sampling. Studies documenting a diurnal pattern used serial sampling of captive individuals, trained for blood collection, whereas our study relied on many individual observations following the stress of a chase capture. A study of captive beluga whales found that the serum cortisol during an exam was approximately 2.4-fold greater than baseline measures, while the greatest diurnal change was only about a 1.4-fold decrease throughout the day [23]. The increase in cortisol following the chase capture in this study was likely greater than the increase observed in captive beluga whales undergoing an exam, therefore it would not be surprising that a diurnal pattern, if it exists, would be masked.

**Table 2:** Linear Mixed Models.

Hormone	N_o_	N_i_	Model Parameters	AIC	R^2^	F	df	p value
**Pre-Sampling Cortisol (**μ**g/dL)**								
Adult Females	48	36	*Age*	192.85	0.31	1.76	1,7	0.23
			***Elapsed Time***			**10.30**	1,7	**0.01**
			*Lactation Status*			0.01	1,7	0.91
			*Time of Day*			0.50	1,7	0.50
			Age*Elapsed Time			3.62	1,7	0.10
Full Model	189	118	*Age*	652.94	0.18	1.94	1, 66	0.17
			***Elapsed Time***			**8.79**	1, 66	**0.004**
			*Sex*			0.01	1, 116	0.94
			*Time of Day*			0.40	1, 66	0.53
			Age*Elapsed Time			0.00	1, 66	0.98
			Sex*Elapsed Time			0.01	1, 66	0.93
Reduced Model	189	118	***Elapsed Time***	611.21	0.13	**28.95**	1, 70	**<0.0001**
**Post-Sampling Cortisol (**μ**g/dL)**								
Full Model	59	52	*Age*	248.83	0.06	1.87	1, 2	0.30
			*Elapsed Time*			0.97	1, 2	0.43
			*Sex*			0.02	1, 50	0.88
			*Time of Day*			0.06	1, 2	0.83
			Age*Elapsed Time			1.50	1, 2	0.35
			Sex*Elapsed Time			0.12	1, 2	0.76
**Pre-Sampling Aldosterone** ^^^								
Full Model	162	103	*Age*	279.68	0.05	0.44	1, 56	0.51
			*Elapsed Time*			2.62	1, 56	0.11
			Age*Elapsed Time			0.01	1, 56	0.90

Linear mixed model (LMM) results for covariates significantly associated with bottlenose dolphin serum concentrations of cortisol and aldosterone. Parameters in bold were significantly associated with cortisol concentrations (p<0.05). Abbreviations: N_o_ = number of observations; N_i_ = number of individuals; F = F statistic; df = numerator, denominator degrees of freedom.

^Log-transformed

**Fig 2:**
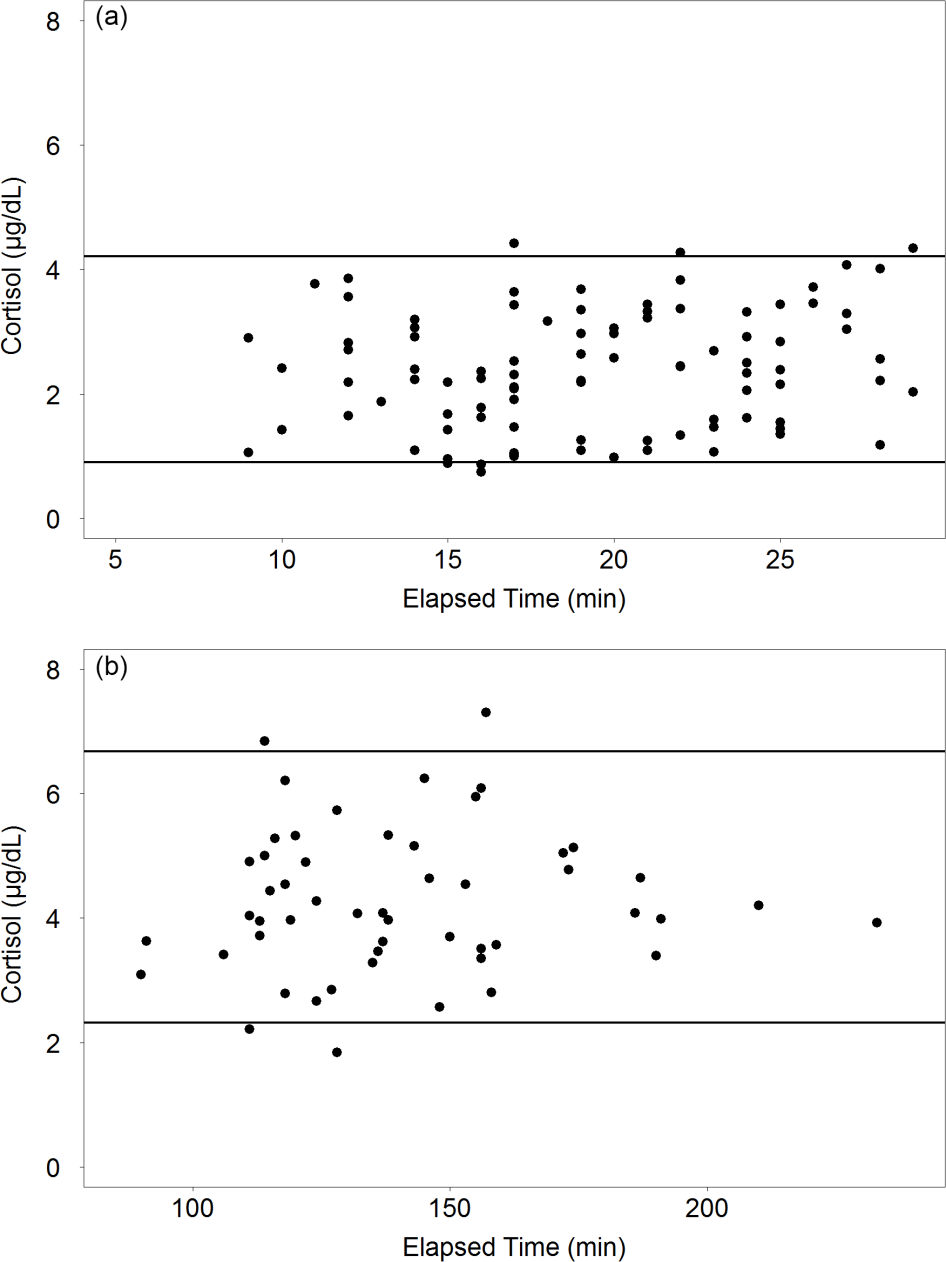
Cortisol Reference Intervals. (a) Pre-sampling (<30 minutes elapsed time) and (b) post-sampling 95^th^ percentile reference intervals for serum cortisol (μg/dL) versus elapsed sampling time (min). Points represent measured pre-sampling *(N = 93)* and post-sampling *(N = 52)* serum cortisol values for bottlenose dolphins sampled in Sarasota Bay, FL 2000–2012; solid lines represent upper and lower bounds for the 95^th^ percentile.

Pre- and post-sampling cortisol concentrations were significantly different (LMM, p<0.0001); therefore, serum cortisol RIs were calculated separately for pre-sampling collection in less than 30 minutes elapsed time (N = 93; Table 3; Fig 2) and post-sampling collection (N = 52; Table 3; Fig 2). Sample sizes for pre-sampling cortisol concentrations collected ≥30 minutes elapsed time (N = 23) were not sufficient for RI calculation [11, 26]. Partitioning analyses, described by Schwacke et al. (2009), confirmed that reference intervals calculated for data stratified by pre- and post-sampling were appropriate (p<0.05). Furthermore, comparisons of these RIs to previous studies suggest the ranges (pre-sampling: 0.91–4.21 μg/dL; post-sampling: 2.32–6.68 μg/dL) to be reasonable as they encompass the mean concentrations and even the range of values reported in other wild bottlenose dolphin serum cortisol studies [7, 22].

**Table 3:** Serum Cortisol Reference Intervals.

Stratum	N	Outliers	95% Reference Interval	95% Reference Interval
			Lower Bound (90% CI)	Upper Bound (90% CI)
**Pre-sampling <30 minutes elapsed**	93	2	0.91 (0.79–1.02)	4.21 (3.81–4.40)
**Post-sampling**	52	0	2.32 (1.84–2.80)	6.68 (5.99–7.30)

Serum cortisol (μg/dL) 95^th^ percentile reference intervals and associated 90% confidence intervals for free-ranging bottlenose dolphins sampled in less than 30 minutes following net deployment (pre-sampling) and dolphins sampled following veterinary procedures (post-sampling).

### Aldosterone

**Fig 3:**
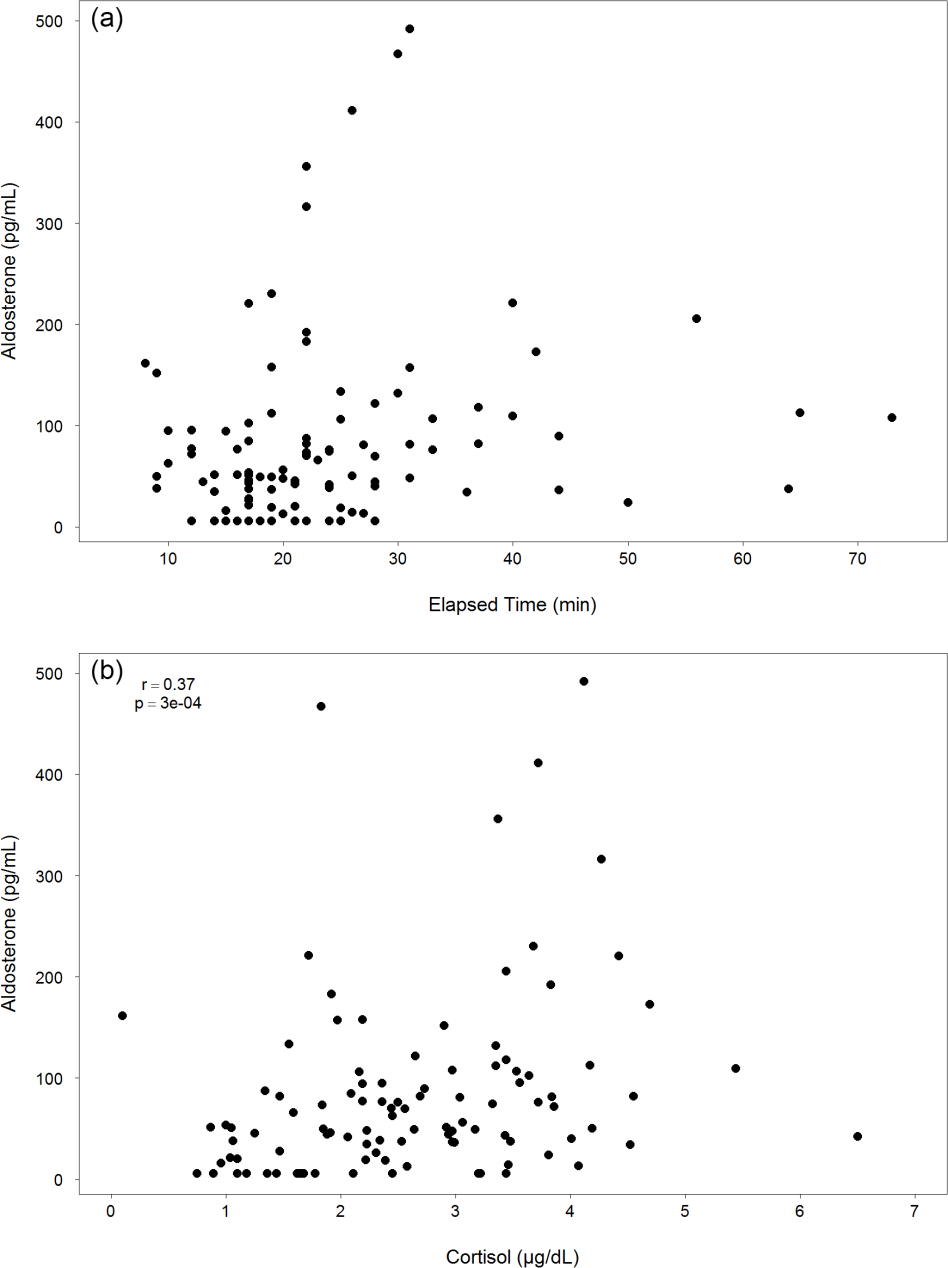
Aldosterone Plots. (a) Pre-sampling aldosterone concentration (pg/mL) versus elapsed sampling time (min) and (b) pre-sampling aldosterone concentration (pg/mL) versus pre-sampling cortisol concentration (μg/dL) for bottlenose dolphins sampled in Sarasota Bay, FL 2000–2012 *(N = 103)*. Correlation coefficient (r) and statistical significance (p) reported for Spearman’s correlation test.

The relationship between aldosterone and elapsed time was similar to cortisol; most samples were collected in less than 30 minutes and there was significant variation among these samples (Fig 3). Previous dolphin studies have found associations between aldosterone and elapsed time [6, 7], as well as significantly higher concentrations among juvenile dolphins compared to adults [22]. Our analyses, however, did not demonstrate these same associations (LMM p>0.05; Table 2). Aldosterone was positively associated with serum cortisol (Spearman’s correlation, r = 0.37; p = 0.0003; Fig 3), which could be indicative of a stress response to capture [4, 7] and is in agreement with previous studies that observed higher concentrations among free-ranging, temporarily restrained bottlenose dolphins compared to those managed under human care [3, 7].

The aldosterone assay detection limit for this study was 11.0 pg/mL, which is as low or lower than the minimum concentration or detection limit previously reported for captive dolphins following a chase-capture scenario (81.7 pg/mL [6]) and free-ranging dolphins captured using methods similar to this study (11.0 pg/mL [22]). However, 16% of the pre-sampling aldosterone concentrations from this study were below the detection limit, making the calculation of a lower 95^th^ percentile RI impractical. While 95^th^ percentiles are commonly accepted for clinical diagnoses of anomalous cases [11, 26], an expected percentage of values below detection limit (e.g. 16%) also provides useful information to identify concentrations that are in the lower portion of the distribution and potentially biologically significant. For example, of the 15 dolphins with aldosterone concentrations below the detection limit (“BDL”), two individuals had cortisol concentrations below the cortisol reference interval and seven were below the 25^th^ percentile. These results suggest that BDL aldosterone could signal concurrent cortisol deficiency, an effect observed among Barataria Bay, LA dolphins suspected to suffer from hypoadrenocorticism following the *Deepwater Horizon* oil spill [14].

### Application

The cortisol RIs developed in this study were based on 95^th^ percentiles; therefore, it would be expected that five percent of random samples from other populations would fall outside of these thresholds. More specifically, 2.5% of values could be expected to fall above and 2.5% below the thresholds for a 95^th^ percentile reference interval. To examine the cortisol RI applicability to other estuarine stocks, we compared serum cortisol concentrations of dolphins sampled at three other study sites (Beaufort, NC; Sapelo/Brunswick, GA; St. Joseph Bay, FL; Table 4) to the computed cortisol RI and calculated the proportion of out of range values. It is important to note that samples were collected into serum separator tubes and processed following similar protocols across the studies, and all samples were analyzed by the same laboratory (Cornell University’s Animal Health Diagnostic Center’s Endocrinology Laboratory).

**Table 4:** Reference Interval Application.

	Beaufort (2000, 2006)	St. Joseph Bay (2005, 2006)	Georgia (2009)
**Sample Sizes**			
Cortisol	4	11	19
Aldosterone	10	28	27
**Cortisol**			
Expected Cases	1 or fewer	2 or fewer	2 or fewer
Observed Cases	0	0	0
Observed Case Prevalence	NA*	0.00	0.00
95% CI	NA*	(0.00–0.28)	(0.00–0.18)
**Aldosterone**			
Expected Cases	4 or fewer	9 or fewer	8 or fewer
Observed Cases	0	5	7
Observed Case Prevalence	0.00	0.18	0.26
95% CI	(0.00–0.31)	(0.06–0.37)	(0.11–0.46)

Number of cases, case prevalence, and sample sizes of bottlenose dolphins sampled in Beaufort, NC (2000, 2006), St. Joseph Bay, FL (2005,2006), and Sapelo/Brunswick GA (2009). “Expected Cases” is the maximum number of dolphins at a given site that would be expected to have either a cortisol concentration below the 95^th^ percentile reference interval or an aldosterone concentration below the assay detection limit, given the sample size and assuming a prevalence of 0.025 (cortisol) or 0.16 (aldosterone). “Observed Cases” is the number of dolphins at a given site that had either a cortisol concentration below the 95^th^ percentile reference interval or an aldosterone concentration below the assay detection limit. “Case Prevalence” was calculated as the ratio of cases to sample size for each hormone. “95% CI” is the 95% binomial confidence interval for the case prevalence. With the exception of Beaufort 2000 data (elapsed time unknown), cortisol sample sizes include animals sampled <30 minutes elapsed time.

*Prevalence not calculated due to small sample size

Beaufort dolphins were sampled in 2000 and 2006 for stock assessment purposes [28]; no health issues were suspected amongst dolphins sampled in either year. Only four non-pregnant dolphins were sampled within 30 minutes of net deployment, and none of their cortisol concentrations were outside of the 95^th^ pre-sampling RI. Although the sample size was small, inhibiting our ability to draw broad conclusions about cortisol concentrations among dolphins from this region, it is noteworthy that all of the cortisol concentrations for these four individuals fell within the RI. Aldosterone measurements were available for 10 dolphins sampled in 2006; none were below the assay detection limit (Table 4).

Of the dolphins sampled near St. Joseph Bay, FL (SJB) in 2005–2006 (Table 4), none had serum cortisol concentrations outside of the RI, while 18% (N = 5) of aldosterone values were BDL. The number of dolphins with BDL aldosterone values was not greater than expected (Table 4). Health assessments were conducted near SJB in 2005 and 2006 in response to an unusual mortality event (UME) attributed to biotoxin exposure [29]. In addition to the detection of domoic acid in tissues, 23% of SJB dolphins had elevated counts of blood eosinophils [13]. Eosinophils have been suggested as a hematological marker of stress, with declines in circulating cells observed following capture events and also the administration of a synthetic form of ACTH (Cortrosyn) [6]. In fact, Thomson and Geraci (1986) observed an inverse relationship between cortisol concentrations and eosinophil counts, where cortisol increased and eosinophils decreased following stimulation. Similar relationships have been observed in California sea lions (*Zalophus californianus*) exposed to domoic acid, where eosinophil counts were higher and cortisol concentrations were lower than in animals not exposed to the algal toxin [30]. These and other mammalian studies have demonstrated that stressful events can lead to cortisol increases and concurrent eosinopenia [31, 32], suggesting that animals with high eosinophil counts could be expected to have low cortisol concentrations [30]. Of the SJB dolphins with high eosinophil counts (N = 7) only one was sampled in <30 minutes elapsed time and excluded from comparisons to cortisol RIs to avoid confounding by the potential interaction between glucocorticoids and eosinophils.

None of the dolphins sampled near Sapelo/Brunswick, GA (SGA) in 2009 had cortisol concentrations above or below the 95^th^ percentile reference interval (Table 4). Seven dolphins (26%) had BDL aldosterone values; the observed count was not greater than the expected count (Table 4). Bottlenose dolphins were sampled near SGA to investigate sublethal health effects resultant from exposure to high concentrations of polychlorinated biphenyl compounds (PCBs) [15]. Findings from the SGA health assessments revealed a negative correlation between PCBs and thyroid hormone measures, as well as T-cell proliferation indices; however, there was no suggestion of adrenal insult [15]. Despite the health issues observed among dolphins sampled near SGA, none had out-of-range cortisol values, and BDL aldosterone counts were not quantitatively different than expected.

Although the cortisol RIs developed in this study were based on data from dolphins sampled in Sarasota Bay, FL, comparison of hormone concentrations from animals sampled at geographically distant sites suggests that the intervals could be applied to other inshore bottlenose dolphin populations. While it is true that two of the comparison populations had health concerns including eosinophilia resultant from biotoxin exposure and high tissue concentrations of PCBs, none of the comparison populations were suspected to have suffered adrenal insult(s). Thus, these animals were appropriate candidates for comparison to the cortisol RI, as it was developed from other dolphins without adrenal issues. Also, the fact that none of the dolphins had cortisol concentrations outside of the RI strengthens the validity of the RI and our assumption of normal adrenal function in these populations. Despite the small sample sizes, these animals, collectively, demonstrate that the RI range of values is appropriate for many individual dolphin stocks. Furthermore, the number of BDL aldosterone concentrations among dolphins sampled from these other sites was not significantly different than expected, which suggests that the detection limit for this particular assay (11 pg/mL) may be reasonable for the identification of individuals with low concentrations, if the 16^th^ percentile is considered acceptably sensitive.

## Conclusions

For free-ranging bottlenose dolphins captured, sampled, and released in Sarasota Bay, FL between 2000 and 2012, pre-sampling serum cortisol concentrations were significantly associated with elapsed time, whereas cortisol concentration collected later (post-sampling, collected after 90 minutes) and pre-sampling aldosterone were not. Cortisol RIs were therefore constructed for different sampling intervals: 1) pre-sampling with elapsed time <30 minutes; and 2) post-sampling (90–273 minutes). The objective of this study was to develop adrenal hormone RIs for the evaluation of an individual’s response to the stress of chase capture. The pre-sampling cortisol RI can be used to identify dolphins that are unable to mount an initial response to a stressor (i.e. a pre-sampling concentration below the RI). The post-sampling RI can be used to determine if a normal response is sustained throughout sampling (i.e. both pre-sampling and post-sampling concentrations within the RI), as well as detect a delayed response (i.e. low pre-sampling concentration but post-sampling concentration within the RI). Due to the large proportion (16%) of Sarasota dolphins with BDL aldosterone concentrations, 95% RIs were not constructed, but although less selective, the detection limit may be useful to identify animals with low aldosterone concentrations. The proportion of BDL aldosterone and out-of-range cortisol concentrations did not exceed expected numbers for any of the other southeastern U.S. sites, thereby suggesting that these thresholds are appropriate for other inshore dolphin populations.

The utility of clinicopathologic RIs has recently been demonstrated by evaluations of hematological and serum chemistry data among individual bottlenose dolphins exposed to anthropogenic pollutants [14, 15] and naturally produced biotoxins [13], as well as body condition profiles for stranded bottlenose dolphins impacted by debilitating interactions with stingrays or fishing gear [33]. Similar to these studies, the reference interval strata for our study were determined using systematic statistical methods, independent observations, and robust sample sizes acquired from health assessment projects spanning more than a decade. Furthermore, we demonstrate that these RIs fit well with samples from other populations of bottlenose dolphins, regardless of sampling site.

Stress hormones play a critical role in the maintenance of homeostasis and physiological acclimation to natural and anthropogenic stressors. Bottlenose dolphin capture-release health assessments have increased in frequency over the past several decades due to questions regarding health effects from harmful algal bloom exposure [13, 34], PCB contamination [15, 35], and in response to environmental disasters such as the *Deepwater Horizon* oil spill [14]. In many cases, baseline health data have not been available for the potentially impacted populations. Reference intervals, based on unbiased statistical analyses and long-term data, provide an opportunity to quantitatively and objectively compare and evaluate health parameters of individuals for which historical data are unavailable [26].

## Disclaimer

This publication does not constitute an endorsement of any commercial product or intend to be an opinion beyond scientific or other results obtained by the National Oceanic and Atmospheric Administration (NOAA). No reference shall be made to NOAA, or this publication furnished by NOAA, to any advertising or sales promotion which would indicate or imply that NOAA recommends or endorses any proprietary product mentioned herein, or which has as its purpose an interest to cause the advertised product to be used or purchased because of this publication.

## Supporting Information

S1 Dataset Health assessment pre- and post-sampling data for bottlenose dolphins (*Tursiops truncatus*) captured and released near Sarasota Bay, FL (2000–2012). Pre-sampling dataset includes: capture-release date, time of net deployment, time that samples were collected, elapsed time (difference in the time from net deployment to sample collection), individual id, sex, age (yrs), pregnancy status, lactation status, serum cortisol concentration (μg/dL), and aldosterone concentration (pg/mL). Post-sampling dataset includes: capture-release date, time of net deployment, time that samples were collected, elapsed time (difference in the time from net deployment to sample collection), individual id, sex, age, pregnancy status, lactation status, and serum cortisol concentration (μg/dL).
(XLSX)
